# Supplementary figures and images for: Effects of DA-9701 (motilitone®) on gastric emptying, glycemic control, and oxidative stress in diabetic rats
Source: PLoS One. 2025 Jun 27;20(6):e0316686. doi: 10.1371/journal.pone.0316686 (PMC12204525; doi:10.1371/journal.pone.0316686)

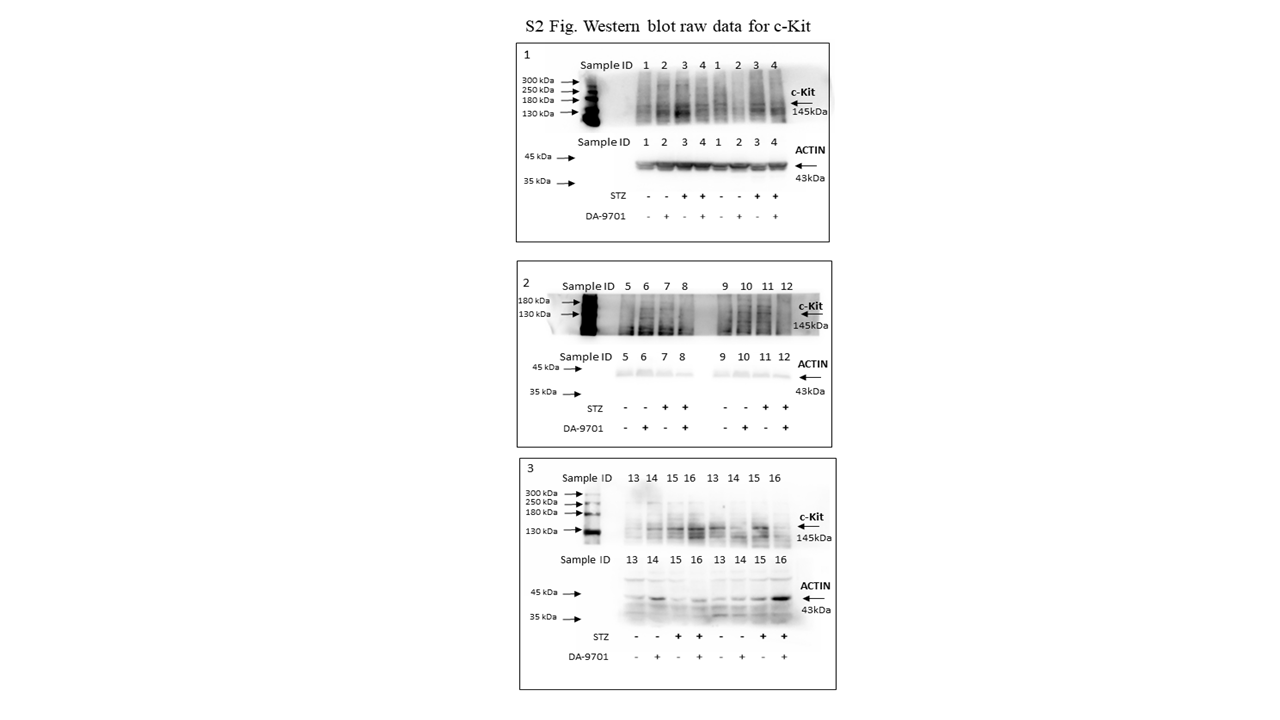

Supplement: S2 Fig — (TIF) [file pone.0316686.s002.tif]

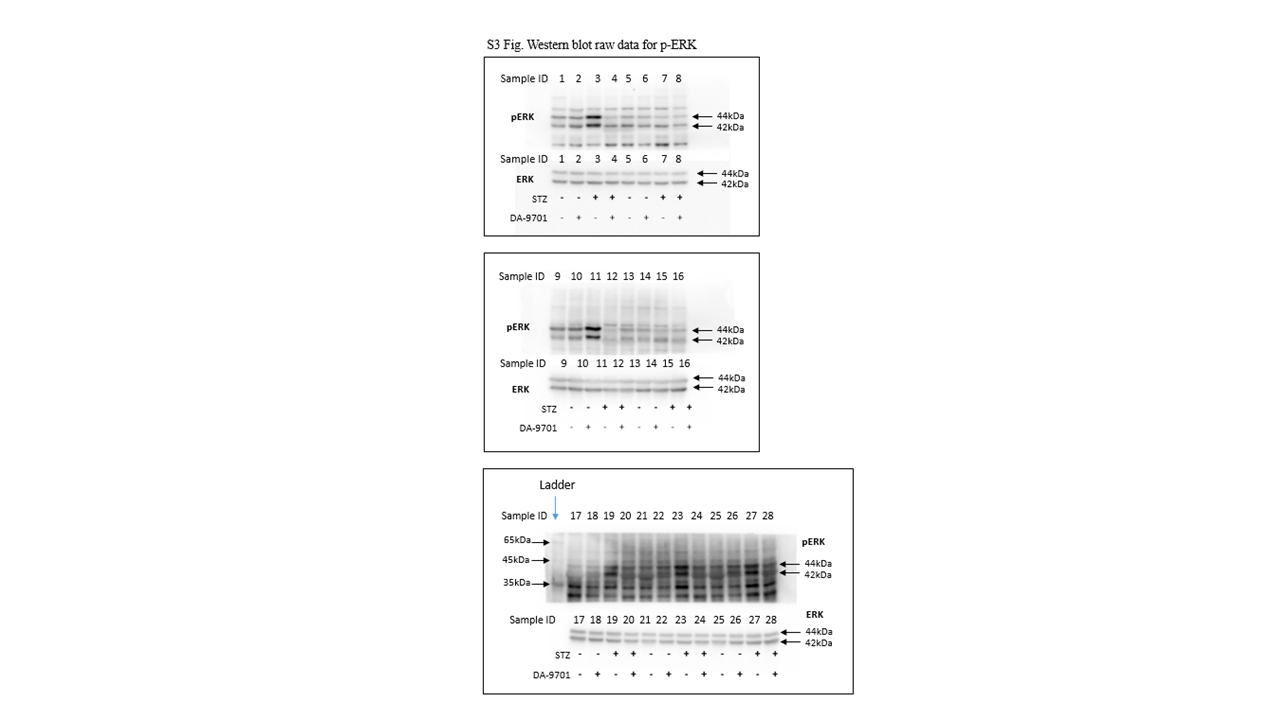

Supplement: S3 Fig — (TIF) [file pone.0316686.s003.tif]

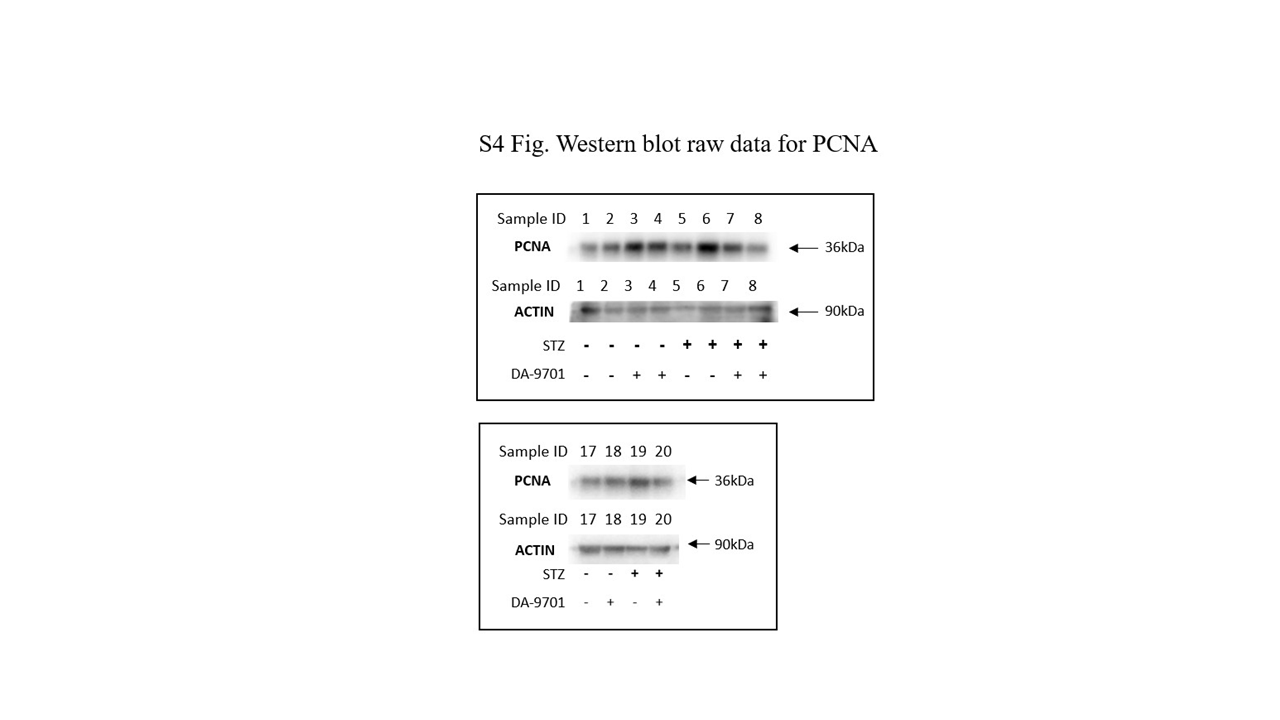

Supplement: S4 Fig — (TIF) [file pone.0316686.s004.tif]

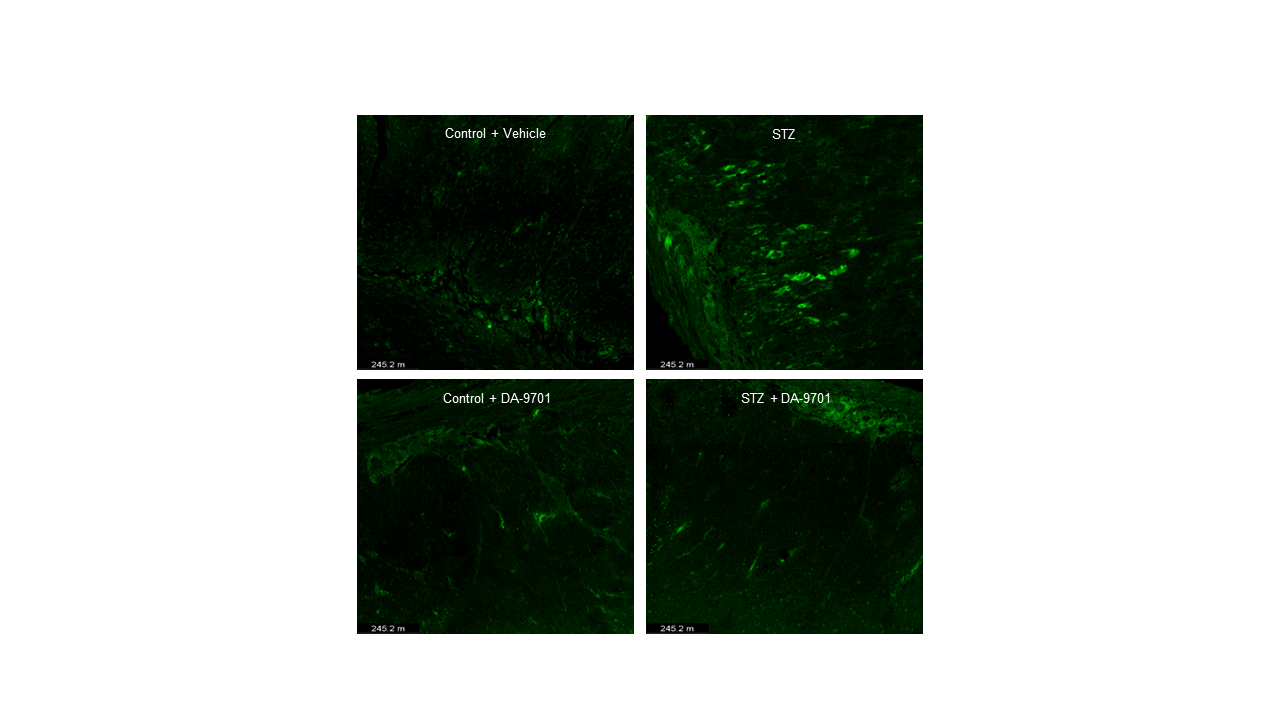

Supplement: S5 Fig — (TIF) [file pone.0316686.s005.tif]
