# Supplementary material for: Effects of DA-9701 (motilitone®) on gastric emptying, glycemic control, and oxidative stress in diabetic rats
Source: PLoS One. 2025 Jun 27;20(6):e0316686. doi: 10.1371/journal.pone.0316686 (PMC12204525; doi:10.1371/journal.pone.0316686)
Supplement: Attachment — Submitted filename: Response to Reviewers.docx [file pone.0316686.s007.docx]

Responses to the Editor’s and Reviewers’ Comments

11 May, 2025

Dear Reviewers and Editorial Staff of *PLOS One*

Thank you for giving me the opportunity to submit a revised draft of our manuscript **“Effects of DA-9701 (motilitone®) on gastric emptying, blood glucose regulation, and oxidative stress in diabetes in rats”.** We appreciate the time and effort that you and the reviewers have dedicated to providing your valuable feedback on our manuscript. We are grateful to the reviewers for their insightful comments on our paper. We have revised the manuscript according to the Reviewer’s suggestions. We have been able to incorporate changes to reflect most of the suggestions provided by the reviewers. We have highlighted (underlined and in blue) the changes within the manuscript.

Here is a point-by-point responses to the reviewers’ comments and concerns.

**Comments from Edtior**

PONE-D-24-54338

Effects of DA-9701 (motilitone®) on gastric emptying, blood glucose regulation, and oxidative stress in diabetes in rats

PLOS ONE

Dear Dr. Sohn,

Thank you for submitting your manuscript to PLOS ONE. After careful consideration, we feel that it has merit but does not fully meet PLOS ONE’s publication criteria as it currently stands. Therefore, we invite you to submit a revised version of the manuscript that addresses the points raised during the review process.


Please submit your revised manuscript by Apr 25 2025 11:59PM. If you will need more time than this to complete your revisions, please reply to this message or contact the journal office at [plosone@plos.org](mailto:plosone@plos.org). When you're ready to submit your revision, log on to https://www.editorialmanager.com/pone/ and select the 'Submissions Needing Revision' folder to locate your manuscript file.

Please include the following items when submitting your revised manuscript:

- A rebuttal letter that responds to each point raised by the academic editor and reviewer(s). You should upload this letter as a separate file labeled 'Response to Reviewers'.
- A marked-up copy of your manuscript that highlights changes made to the original version. You should upload this as a separate file labeled 'Revised Manuscript with Track Changes'.
- An unmarked version of your revised paper without tracked changes. You should upload this as a separate file labeled 'Manuscript'.

If you would like to make changes to your financial disclosure, please include your updated statement in your cover letter. Guidelines for resubmitting your figure files are available below the reviewer comments at the end of this letter.

If applicable, we recommend that you deposit your laboratory protocols in protocols.io to enhance the reproducibility of your results. Protocols.io assigns your protocol its own identifier (DOI) so that it can be cited independently in the future. For instructions see: <https://journals.plos.org/plosone/s/submission-guidelines#loc-laboratory-protocols>. Additionally, PLOS ONE offers an option for publishing peer-reviewed Lab Protocol articles, which describe protocols hosted on protocols.io. Read more information on sharing protocols at <https://plos.org/protocols?utm_medium=editorial-email&utm_source=authorletters&utm_campaign=protocols>.

We look forward to receiving your revised manuscript.

Kind regards,

Rajesh Kumar Singh, Ph.D.
Academic Editor
PLOS ONE

Journal requirements:

When submitting your revision, we need you to address these additional requirements.

1. Please ensure that your manuscript meets PLOS ONE's style requirements, including those for file naming. The PLOS ONE style templates can be found at

https://journals.plos.org/plosone/s/file?id=wjVg/PLOSOne_formatting_sample_main_body.pdf and

<https://journals.plos.org/plosone/s/file?id=ba62/PLOSOne_formatting_sample_title_authors_affiliations.pdf>

**Author’s response**: We sincerely appreciate your helpful guidance regarding the journal’s formatting requirements. In accordance with the PLOS ONE style guidelines, we have carefully revised the manuscript, including the formatting of the author names (such as the inclusion of commas between authors), refinement of institutional affiliations (including removal of redundant location names), and an update to reflect a recent institutional affiliation change due to the corresponding author's new appointment.

To facilitate your review, all changes have been clearly indicated using tracked changes, and the revised content has been highlighted in yellow at lines 5 and 10–11. The updated file has been submitted under the filename “Track changes version of the manuscript.docx.” We hope that the revised version now meets all formatting requirements of the journal.

2. We note that your Data Availability Statement is currently as follows: [All relevant data are within the manuscript and its Supporting Information files.]

Please confirm at this time whether or not your submission contains all raw data required to replicate the results of your study. Authors must share the “minimal data set” for their submission. PLOS defines the minimal data set to consist of the data required to replicate all study findings reported in the article, as well as related metadata and methods (https://journals.plos.org/plosone/s/data-availability#loc-minimal-data-set-definition).

For example, authors should submit the following data:

- The values behind the means, standard deviations and other measures reported;

- The values used to build graphs;

- The points extracted from images for analysis.

Authors do not need to submit their entire data set if only a portion of the data was used in the reported study.

If your submission does not contain these data, please either upload them as Supporting Information files or deposit them to a stable, public repository and provide us with the relevant URLs, DOIs, or accession numbers. For a list of recommended repositories, please see https://journals.plos.org/plosone/s/recommended-repositories.

If there are ethical or legal restrictions on sharing a de-identified data set, please explain them in detail (e.g., data contain potentially sensitive information, data are owned by a third-party organization, etc.) and who has imposed them (e.g., an ethics committee). Please also provide contact information for a data access committee, ethics committee, or other institutional body to which data requests may be sent. If data are owned by a third party, please indicate how others may request data access.

**Author’s response**: We sincerely appreciate the opportunity to revise our manuscript and fully acknowledge the importance of data transparency. Accordingly, we will provide all relevant raw data as a Supporting Information file titled “**S1 Raw data for body weight, blood glucose, gastric emptying, oxidative stress markers, and Western blot analyses**” in our revised submission, in compliance with the journal’s data availability policy.

3. PLOS ONE now requires that authors provide the original uncropped and unadjusted images underlying all blot or gel results reported in a submission’s figures or Supporting Information files. This policy and the journal’s other requirements for blot/gel reporting and figure preparation are described in detail at https://journals.plos.org/plosone/s/figures#loc-blot-and-gel-reporting-requirements and https://journals.plos.org/plosone/s/figures#loc-preparing-figures-from-image-files. When you submit your revised manuscript, please ensure that your figures adhere fully to these guidelines and provide the original underlying images for all blot or gel data reported in your submission. See the following link for instructions on providing the original image data: https://journals.plos.org/plosone/s/figures#loc-original-images-for-blots-and-gels.

In your cover letter, please note whether your blot/gel image data are in Supporting Information or posted at a public data repository, provide the repository URL if relevant, and provide specific details as to which raw blot/gel images, if any, are not available. Email us at plosone@plos.org if you have any questions.

**Author’s response**: We sincerely thank the editor and reviewers for their valuable comments regarding the figure preparation and blot/gel data reporting.

In full compliance with the PLOS ONE guidelines, we will provide all original, uncropped, and unadjusted blot and gel images associated with the results presented in the manuscript. These images will be compiled into Supporting Information files titled “**S2 Fig Western blot raw data for c-Kit, S3 Fig Western blot raw data for p-ERK/t-ERK, and S4 Fig Western blot raw data for PCNA**”, and will be submitted alongside the revised manuscript.

Each image will be clearly annotated to indicate sample identity, loading order, molecular weight markers, and the corresponding figure panels, in accordance with the journal’s standards. Any non-included lanes will be marked appropriately, and no modifications will be made that could compromise data integrity.

We greatly appreciate the journal’s emphasis on transparency and reproducibility, and are pleased to ensure that our figures fully adhere to these important standards.

Additional Editor Comments:

The manuscript entitled "Effects of DA-9701 (motilitone®) on gastric emptying, blood glucose regulation, and oxidative stress in diabetes in rats" needs revision as suggested by the reviewers.


[Note: HTML markup is below. Please do not edit.]

Reviewers' comments:

Reviewer's Responses to Questions

**Comments to the Author**

1. Is the manuscript technically sound, and do the data support the conclusions?

The manuscript must describe a technically sound piece of scientific research with data that supports the conclusions. Experiments must have been conducted rigorously, with appropriate controls, replication, and sample sizes. The conclusions must be drawn appropriately based on the data presented.

Reviewer #1: Yes

Reviewer #2: Partly

2. Has the statistical analysis been performed appropriately and rigorously?

Reviewer #1: No

Reviewer #2: Yes

3. Have the authors made all data underlying the findings in their manuscript fully available?

The [PLOS Data policy](http://www.plosone.org/static/policies.action#sharing) requires authors to make all data underlying the findings described in their manuscript fully available without restriction, with rare exception (please refer to the Data Availability Statement in the manuscript PDF file). The data should be provided as part of the manuscript or its supporting information, or deposited to a public repository. For example, in addition to summary statistics, the data points behind means, medians and variance measures should be available. If there are restrictions on publicly sharing data—e.g. participant privacy or use of data from a third party—those must be specified.

Reviewer #1: Yes

Reviewer #2: Yes

4. Is the manuscript presented in an intelligible fashion and written in standard English?

PLOS ONE does not copyedit accepted manuscripts, so the language in submitted articles must be clear, correct, and unambiguous. Any typographical or grammatical errors should be corrected at revision, so please note any specific errors here.

Reviewer #1: Yes

Reviewer #2: Yes

5. **Review Comments to the Author**

Please use the space provided to explain your answers to the questions above. You may also include additional comments for the author, including concerns about dual publication, research ethics, or publication ethics. (Please upload your review as an attachment if it exceeds 20,000 characters)

**Comments from Reviewer #1:**

1) *Sample sizes (4-6 per group) are relatively small, though adequate for statistical analysis. The study duration of 4 weeks may be insufficient to fully evaluate long-term effects.*

**Author’s response**: We appreciate the reviewer's comment. You have raised an important point here. We aimed to verify whether early-stage alterations in gastric emptying and the functional dynamics within the ICC networks occur in a Type 1 diabetic rat model. However, since STZ (65 mg/kg) administration induces early-stage diabetes within days to weeks-characterized by rapid metabolic disturbances and hyperglycemia- this model's rapid disease progression limited our ability to evaluate long-term effects. (Reference: Duration of streptozotocin-induced diabetes differentially affects p38-mitogen-activated protein kinase (MAPK) phosphorylation in renal and vascular dysfunction. Cardiovasc Diabetol. 2005 Mar 5:4:3.doi: 10.1186/1475-2840-4-3.)

2) *Include mechanistic investigation of DA-9701's effect on insulin secretion.*

**Author’s response**:

Although the increase was not statistically significant, we observed a trend toward increased pancreas weight in the Corydalis-treated group compared to the STZ group. This may suggest that Corydalis inhibits STZ-induced β-cell apoptosis, thereby enhancing insulin secretion. To investigate this potential mechanism, we are currently conducting further studies using STZ-induced diabetic mice.

3) *Provide more detailed histological analysis of ICC networks*

STZ + Vehicle

Control + Vehicle

Control + DA-9701

STZ + DA-9701

**Author’s response**: We appreciate the reviewer’s comment. In response to the suggestion, we performed immunostaining using c-KIT antibody.

4) *Consider adding long-term follow-up data*

**Author’s response:** Thank you for this valuable suggestion. We acknowledge the importance of long-term follow-up data in fully understanding the progression of diabetes-related changes. However, as mentioned in our response to the previous comment, the rapid disease progression in the STZ-induced diabetic rat model limits our ability to conduct long-term studies. We have added a discussion of this limitation to the manuscript, highlighting both the strengths and constraints of our chosen model for studying early-stage alterations. We also suggest potential future directions for addressing long-term effects using alternative models or approaches.

5) *Include dose-response studies for DA-9701.*

**Author’s response:** Thank you for your suggestion to include dose-response studies for DA-9701. We acknowledge that several studies have demonstrated dose-dependent effects of DA-9701, particularly in enhancing gastric motility and improving visceral hypersensitivity. However, our study focused on verifying the efficacy of DA-9701 at a clinically relevant dose rather than establishing a dose-response relationship.

We calculated the dose for our rat model based on the standard therapeutic dose used in humans, adjusting for metabolic rate differences between species. This approach allowed us to directly translate our findings to clinical applications. While we recognize the value of dose-response studies, our primary objective was to assess the effectiveness of DA-9701 at a dose that mirrors current human usage.

Future studies could indeed explore the dose-dependent effects of DA-9701 in our specific model, which could provide additional insights into its mechanism of action and potential optimization of dosing regimens.

6) *Add error bars to all figures where missing.*

**Author’s response:** We appreciate the reviewer's comment. We have carefully re-examined the original data and the graphs. As confirmed from the raw data, the variability among individual values within the control group was relatively small compared to the scale of the Y-axis. Therefore, the error bars in the control group appeared very short and were not readily visible in the previous figures.

The graphs were generated using GraphPad Prism software, which automatically plots error bars. Given this, it is unlikely that the error bars were accidentally omitted in any part of the graphs.

For further clarification, we plotted the control group data separately with a reduced Y-axis range. As shown in the attached graph, small error bars can now be observed, confirming that the error bars were originally present but not easily visible due to the Y-axis scale.

7) *Provide more detailed methods for insulin measurement*

**Author’s response:** We appreciate the reviewer's comment. In response to the suggestion, we have added detailed methods for insulin measurement to the Material and Methods section as shown below:

To comply with animal protocol guidelines on blood volume collection, we collected blood samples from mice at 0, 15, 30, and 60 minutes following glucose administration using heparin-coated capillary microvette® tubes (SARSTEDT, 20.1282.100) to measure plasma insulin levels. After centrifugation at 13,000 rpm for 5 minutes at 4°C, plasma was isolated and stored at -80°C until further analysis. Plasma insulin levels were quantified using an ELISA kit (Alpoco Diagnostics, Salem, NH, USA), according to the manufacturers’ instructions.

To measure serum malondialdehyde (MDA) levels, blood was collected via cardiac puncture at the time of euthanasia and centrifuged at 13,000 rpm for 5 minutes at 4°C. Serum MDA levels were measured using a lipid peroxidation assay kit, following the manufacturers’ instructions (Abcam, Cambridge, United Kingdom).

8) *Include additional controls for oxidative stress measurements*

**Author’s response:** We appreciate the reviewer's comment. In response to the suggestion, we performed real-time PCR using oxidative stress-related genes (*Gpx* and *Cat*) and added these results to Fiugre 5, which is shown below;

9) *Clarify the timing of measurements in the experimental timeline*

**Author’s response:** We appreciate the reviewer's comment. We indicated the time points of blood collection for insulin measurement in the schema of the experimental schedule.

**Comments from Reviewer #2: Comment to Author**
The manuscript offers valuable insights into the early diabetic complications of accelerated gastric emptying and demonstrates the potential of DA-9701 in normalizing gastric motility and improving glycemic control. However, there are a few possibilities to improve the manuscript, which are listed below.
Title-
The revised title eliminates redundancy and improves readability.
Suggested Title: "Effects of DA-9701 (Motilitone®) on Gastric Emptying, Glycemic Control, and Oxidative Stress in Diabetic Rats"
 **Author’s response:** We sincerely thank the reviewer for the insightful and constructive comment regarding the manuscript title. We fully agree that the proposed revision enhances both clarity and conciseness by eliminating redundancy and improving readability. In accordance with the suggestion, we have revised the title as follows:

"Effects of DA-9701 (Motilitone®) on Gastric Emptying, Glycemic Control, and Oxidative Stress in Diabetic Rats"

The revised title has been highlighted with a yellow background in the track-changed version of the manuscript for the reviewer’s convenience.

We are grateful for the reviewer’s thoughtful recommendation, which has contributed to the refinement and overall quality of the manuscript.

**Abstract:**
1) Lacks numerical data, making it less informative. Include key statistical outcomes to strengthen the impact. Consider summarizing mechanisms more explicitly. For example, how DA-9701 affects oxidative stress pathways. Clarify if DA-9701 showed a dose-dependent effect or if a single dose was used.

**Author’s response:** We sincerely thank the reviewer for the constructive feedback on our abstract. In response, we have implemented the following key revisions to enhance clarity, scientific impact, and mechanistic detail:

Incorporation of Numerical Data:

We added specific quantitative results to substantiate our conclusions. These include a three- to four-fold increase in fasting blood glucose levels in diabetic rats compared to controls (p < 0.001), a ~25% reduction in OGTT AUC following DA-9701 treatment (p < 0.01), and a 2.1-fold increase in plasma insulin secretion (p < 0.05). These data help to quantify the physiological relevance of our findings.

Expanded Mechanistic Description:

DA-9701 administration significantly reduced oxidative stress in diabetic gastric tissue, as evidenced by decreased levels of MDA and Ogg1, both established markers of lipid peroxidation and oxidative DNA damage. In addition, DA-9701 suppressed the compensatory upregulation of antioxidant-related genes, including glutathione peroxidase (Gpx) and catalase (Cat), which are typically elevated in response to oxidative stress. These molecular changes were accompanied by reduced phosphorylation of ERK1/2 and decreased expression of c-Kit and PCNA, which are associated with interstitial cell activity and cellular remodeling.

Clarification of Dosing Information:

We acknowledge that several studies have demonstrated dose-dependent effects of DA-9701, particularly in enhancing gastric motility and improving visceral hypersensitivity. However, our study focused on verifying the efficacy of DA-9701 at a clinically relevant dose rather than establishing a dose-response relationship.

We calculated the dose for our rat model based on the standard therapeutic dose used in humans, adjusting for metabolic rate differences between species. This approach allowed us to directly translate our findings to clinical applications. While we recognize the value of dose-response studies, our primary objective was to assess the effectiveness of DA-9701 at a dose that mirrors current human usage.

Future studies could indeed explore the dose-dependent effects of DA-9701 in our specific model, which could provide additional insights into its mechanism of action and potential optimization of dosing regimens.

These revisions have been incorporated into the abstract and are clearly marked in the tracked version of the manuscript (lines 43–51).

We hope that these changes meet the reviewer’s expectations and improve the clarity and rigor of the manuscript.

**Background/Aims (Lines 28-35)**
• Line 29: "These alterations can significantly influence gastrointestinal symptoms and potentially impact blood glucose levels."-Suggest replacing "potentially impact" with "directly influence" for stronger phrasing.

**Author’s response:** We appreciate the your insightful comment regarding the phrasing in line 29. As suggested, we have replaced “potentially impact” with “directly influence” to strengthen the statement and better reflect the established relationship between gastric motility and glycemic control. The revised sentence now reads:

“These alterations can significantly influence gastrointestinal symptoms and directly influence blood glucose levels.”

This change has been incorporated into the revised manuscript and is highlighted accordingly in the tracked version.

• Lines 31-32: The function of DA-9701 is well-stated, but a brief mention of its specific active compounds here would provide more context.

**Author’s response:** We appreciate your thoughtful suggestion to include a brief mention of the active compounds in DA-9701. We fully agree that such information enhances the pharmacological context of the agent. While we have not added this detail directly to the Background/Aims section of the abstract in order to maintain conciseness, we respectfully note that the specific active constituents of DA-9701 such as *Corydalis* tuber and *Pharbitis* seed are described in the Introduction section (line 30), where their prokinetic, antiulcer, and analgesic properties are discussed in more detail.

Should the reviewer recommend its inclusion in the abstract as well, we would be happy to revise accordingly.

• Line 33-34: Instead of "validating its broader clinical utility," consider "assessing its potential therapeutic application."

**Author’s response:** We thank for the helpful suggestion to improve the clarity and precision of our phrasing. In accordance with the comment, we have replaced “validating its broader clinical utility” with “assessing its potential therapeutic application” to more accurately reflect the scope of the study. The revised sentence now reads:

“...to investigate the potential of DA-9701 in addressing altered gastric emptying and glycemic control in diabetic rats, thereby assessing its potential therapeutic application.”

This revision has been made in the updated manuscript and is visible in the tracked changes version.

• Line 47: "Hyperglycemia-induced oxidative stress" should be briefly explained with supporting evidence.

**Author’s response:** We thank you for the valuable suggestion to provide a clearer explanation of the term “hyperglycemia-induced oxidative stress” in the Results section of Abstract. As recommended, we have revised the abstract to include a concise mechanistic description. Specifically, we now state that hyperglycemia is known to enhance reactive oxygen species production and activate the ERK signaling pathway, which in turn upregulates c-Kit expression and contributes to ICC hyperactivity and accelerated gastric emptying. This mechanistic link is supported by both our experimental data (elevated MDA, Ogg1, and p-ERK) and previous literature (e.g., Goyal RK. Gastric Emptying Abnormalities in Diabetes Mellitus. N Engl J Med. 2021;384(18):1742-51., Hayashi Y, Toyomasu Y, Saravanaperumal SA, Bardsley MR, Smestad JA, Lorincz A, et al. Hyperglycemia Increases Interstitial Cells of Cajal via MAPK1 and MAPK3 Signaling to ETV1 and KIT, Leading to Rapid Gastric Emptying. Gastroenterology. 2017;153(2):521-35.e20.). This addition has been incorporated into the revised Results section of the abstract and is highlighted in the tracked manuscript.

**Materials/Methods:**

1) *The description is clear but lacks details on the number of animals per group for different analyses. Consider adding a justification for the 6 mg/kg dose.*

**Author’s response:** The dosage of Corydalis administered to mice was calculated based on a human-to-animal dose conversion method, as previously reported.

Reference: A simple practice guide for dose conversion between animals and human. J Basic Clin Pharm 2016;7:27–31.

2) *Describe why acetaminophen absorption is used as a proxy for gastric emptying. Clarify the relevance of solid vs. liquid gastric emptying assessments.*

**Author’s response:**

1. Acetaminophen absorption is widely used as a proxy measure for gastric emptying because acetaminophen itself is minimally absorbed in the stomach but is rapidly absorbed once it reaches the small intestine. Therefore, the rate of appearance and the time-to-peak concentration of acetaminophen in the blood directly reflect the rate of gastric emptying.
2. Relevance of Solid vs. Liquid Gastric Emptying Assessments

- Solid Gastric Emptying Assessment: Solid gastric emptying assessments are known to be more sensitive for diagnosing gastroparesis. This is because solid foods remain in the stomach for a longer period.
- Liquid Gastric Emptying Assessment: Liquid gastric emptying should be evaluated separately from solid emptying. Some patients may have normal solid gastric emptying but delayed liquid emptying, in which case liquid assessments can provide additional diagnostic value.
- Therefore, evaluating both solid and liquid gastric emptying provides a more comprehensive understanding of gastric motility. Liquid gastric emptying can help detect delays that might not be identified through solid assessments alone.

**Result & Discussion**
The study primarily focuses on short-term effects (2–4 weeks). It remains unclear whether DA-9701 provides sustained benefits or if compensatory mechanisms might alter its efficacy over time.

**Author’s response:** Thank you for this valuable suggestion. We acknowledge the importance of long-term follow-up data in fully understanding the progression of diabetes-related changes. However, as mentioned in our response to the previous comment, the rapid disease progression in the STZ-induced diabetic rat model limits our ability to conduct long-term studies. We have added a discussion of this limitation to the manuscript, highlighting both the strengths and constraints of our chosen model for studying early-stage alterations. We also suggest potential future directions for addressing long-term effects using alternative models or approaches.
 While c-Kit expression and ERK1/2 activation are discussed, the study does not fully investigate the downstream signaling pathways or alternative mechanisms that could contribute to the observed changes in gastric motility.

**Author’s response:** We appreciate the reviewer’s insightful comment. In response to the reviewer’s suggestion, we conducted additional experiments to evaluate the and the ERK1/2 and c-KIT downstream signaling pathways.

Previous studies have reported that hyperglycemia can induce hyperplasia of ICCs, and phosphorylated ERK (p-ERK) is known to promote PCNA expression (reference; Hyperglycemia Increases Interstitial Cells of Cajal via MAPK1 and MAPK3 Signaling to ETV1 and KIT, Leading to Rapid Gastric Emptying, Nat Commun. 2022 Dec 3;13:7476. doi: 10.1038/s41467-022-35230-4). Based on these findings, we assessed PCNA expression levels in our study. Interestingly, STZ treatment resulted in increased PCNA expression. This elevation in PCNA expression may suggest an increase in the number of ICCs expressing c-KIT.

The study does not compare DA-9701 with other established treatments for gastric motility disorders or diabetes-related gastroparesis, limiting its clinical relevance.

**Author’s response:**

Mosapride has been found to decrease blood glucose and increase insulin secretion in normal rats during glucose tolerance tests. This effect is associated with increased glucose-stimulated insulin secretion, which is abolished by a 5-HT4 receptor antagonist.

While DA-9701 improved glucose levels and insulin secretion, the study does not clarify whether this effect is due to direct pancreatic stimulation, altered gastric emptying, or other metabolic factors.
**Author’s response:**

Although the increase was not statistically significant, we observed a trend toward increased pancreas weight in the Corydalis-treated group compared to the STZ group. This may suggest that Corydalis inhibits STZ-induced β-cell apoptosis, thereby enhancing insulin secretion. To investigate this potential mechanism, we are currently conducting further studies using STZ-induced diabetic mice.

Only MDA and Ogg1 are assessed. Including additional oxidative stress markers (e.g., SOD, CAT, GSH) would provide a more comprehensive picture of DA-9701's antioxidant effects.

6. PLOS authors have the option to publish the peer review history of their article ([what does this mean?](https://journals.plos.org/plosone/s/editorial-and-peer-review-process#loc-peer-review-history)). If published, this will include your full peer review and any attached files.


If you choose “no”, your identity will remain anonymous but your review may still be made public.


**Do you want your identity to be public for this peer review?** For information about this choice, including consent withdrawal, please see our [Privacy Policy](https://www.plos.org/privacy-policy).

Reviewer #1: No

Reviewer #2: **Yes:** Ayush Kumar Garg
